# Native Mass Spectrometry Coupled to Spectroscopic Methods to Investigate the Effect of Soybean Isoflavones on Structural Stability and Aggregation of Zinc Deficient and Metal-Free Superoxide Dismutase

**DOI:** 10.3390/molecules27217303

**Published:** 2022-10-27

**Authors:** Xinyu Bian, Xiaoyu Zhuang, Junpeng Xing, Shu Liu, Zhiqiang Liu, Fengrui Song

**Affiliations:** 1 State Key Laboratory of Electroanalytical Chemistry & Jilin Province Key Laboratory of Chinese Medicine Chemistry and Mass Spectrometry, Changchun Institute of Applied Chemistry, Chinese Academy of Sciences, Changchun 130022, China; 2 School of Applied Chemistry and Engineering, University of Science and Technology of China, Hefei 230029, China; 3 Experiment Center for Science and Technology, Shanghai University of Traditional Chinese Medicine, Shanghai 201203, China

**Keywords:** superoxide dismutase, isoflavones, ion mobility-mass spectrometry, amyotrophic lateral sclerosis, aggregation

## Abstract

The deficiency or wrong combination of metal ions in Cu, Zn-superoxide dismutase (SOD1), is regarded as one of the main factors causing the aggregation of SOD1 and then inducing amyotrophic lateral sclerosis (ALS). A ligands-targets screening process based on native electrospray ionization ion mobility mass spectrometry (ESI-IMS-MS) was established in this study. Four glycosides including daidzin, sophoricoside, glycitin, and genistin were screened out from seven soybean isoflavone compounds and were found to interact with zinc-deficient or metal-free SOD1. The structure and conformation stability of metal-free and zinc-deficient SOD1 and their complexes with the four glycosides was investigated by collision-induced dissociation (CID) and collision-induced unfolding (CIU). The four glycosides could strongly bind to the metal-free and copper recombined SOD1 and enhance the folding stability of these proteins. Additionally, the ThT fluorescence assay showed that these glycosides could inhibit the toxic aggregation of the zinc-deficient or metal-free SOD1. The competitive interaction experiments together with molecular docking indicate that glycitin, which showed the best stabilizing effects, binds with SOD1 between β-sheet 6 and loop IV. In short, this study provides good insight into the relationship between inhibitors and different SOD1s.

## 1. Introduction

Cu, Zn-superoxide dismutase (SOD1, a 32 kDa homodimeric protein) was the first protein found to be closely related to amyotrophic lateral sclerosis (ALS) [1]. One of the possible mechanisms of ALS is the death of motor neurons caused by toxic aggregated SOD1. The toxic aggregated SOD1 has been found in both familial and sporadic ALS cases [2]. The misfolding and dissociation of SOD1 due to mutations, wrong metallization, or aberrant post-translational modifications are the main causes of aggregation [3,4,5].

Although there is still no cure strategy for ALS, researchers never stop making efforts to find effective medicine and treatment methods. Inhibiting the aggregation of SOD1 was thought to be one of the key treatment methods [6]. Some small molecules or peptides that contain high-affinity structures with target proteins may disturb or change the aggregation pathway [7,8,9]. A variety of such molecules were searched from natural products [10,11,12]. In our previous work, some natural flavonoids and their derivatives have been studied, and proved to be effective inhibitors of SOD1 misfolding and aggregation [13,14,15,16].

Soybean isoflavones including aglycones and glycosides are a series of 3-benzopyranone compounds mainly found in soybeans [17]. Epidemiological investigations and research have connected these compounds to a variety of health factors such as limiting the development of cardiovascular diseases, osteoporosis, type 2 diabetes, and breast cancer [18,19,20,21]. The ability of antioxidants, the regulation of hormones, and the cell growth of isoflavones lead to the reduction of disease risk [22,23]. Many studies have proved the antioxidant properties of soybean isoflavones by the expression and activity changes of SOD1 [22]. However, no research has ever been concerned about the possible direct influence on the structure of SOD1 taken by these isoflavones.

Native mass spectrometry (MS) has been proven effective in analyzing the structural dynamics of non-covalent protein interactions such as protein-ligand (P-L) interactions [24]. The binding situation of P-L can be read directly from mass to charge (*m*/*z*) changes in the spectra. The signal intensity of the complexes usually provides binding affinity information of ligands. Comparing the structure stability of protein and P-L complexes is a reliable way of screening more targeted drug molecules [25]. Sometimes the research of ligand binding requires denaturing of the protein in order to increase the sensitivity or fragmentation of the protein to study the binding sites. This would reduce the biological significance. Ion mobility spectrometry can show structure information in the gas phase while keeping the native state in the solution during sample pretreatment [26]. Additionally, collision-induced-dissociation and collision-induced-unfolding are two adjunctive methods by giving collision energy (CE) to proteins or P-L complexes. The resulting structural changes bring different drift times (DT) and peak intensity distributions in ion mobility spectrometry (IMS) [27].

Based on our previous work, some kinds of flavonoids [13], stilbenoids [14], catechins [15], and caffeoylquinic acids [16] were found to form stable non-covalent complexes with wild-type SOD1 (Cu_2_Zn_2_SOD1) or metal-free SOD1 (ApoSOD1). Some of these compounds were proved to inhibit the aggregation of ApoSOD1. As isoflavones contain similar structure as the compounds mentioned above and show multiple biological functions, in this study, we firstly used the ESI-MS method to screen the bioactive ligands, which could effectively interact with different SOD1s from the incubation system of the soybean isoflavone mixture with various SOD1 species and confirm the target SOD1 species of these bioactive ligands (four glycosides). We then investigated the interaction between bioactive isoflavone glycoside and target SOD1s. Partly or wrongly metallated SOD1 has been reported to induce the toxic aggregation linked with ALS [28]. Therefore, two kinds of abnormally metallated SOD1s became our targets for screening, interaction, structure, and aggregation analysis. CID and CIU experiments were performed to show the influence on the target SOD1s’ structure taken by the isoflavone glycosides. The method of TFE-induced aggregation was used to investigate the inhibition ability of the isoflavone glycosides to the aggregation of target SOD1s. The binding sites of these bioactive ligands on target SOD1s were predicted by experimental and theoretical methods.

## 2. Results and Discussion

### 2.1. Screening of Potential Inhibitors and Targets

**Figure 1:**
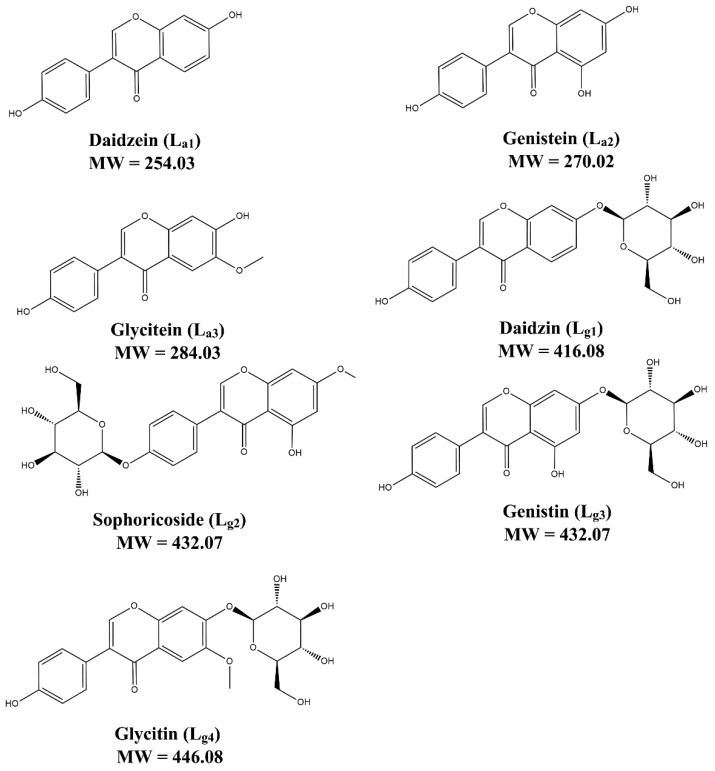
The structure of seven soybean isoflavones.

Ligands that can bind with SOD1 are firstly screened from a mixture of seven soybean isoflavones, including three aglycones (L_a1_–L_a3_) and four glycosides (L_g1_–L_g4_). Their structures are shown in Figure 1. ESI–MS experiments of the isoflavone mixture with and without ApoSOD1, Cu_2_SOD1, and Cu_2_Zn_2_ SOD1 (P_1_, P_2_, and P_3_) are performed. Here we used bovine SOD1 instead of human SOD1, as their secondary structure are quite similar (referred from 1SXA and 6FN8 in the PDB database). Observing the MS intensity fading of free components is a usual method in ligand screening [29]. Here, compared with the intensity fading mass spectra (Figure S1, Table S1), it can be found that the intensity of free L_g1_–L_g4_ decreases after the addition of proteins while the intensity of free L_a1_–L_a3_ does not change the attenuation of free L_g1_–L_g4_, which indicates that part of these glycosides interact with the SOD1s. Thus, the following study focuses on these glycosides.

**Figure 2:**
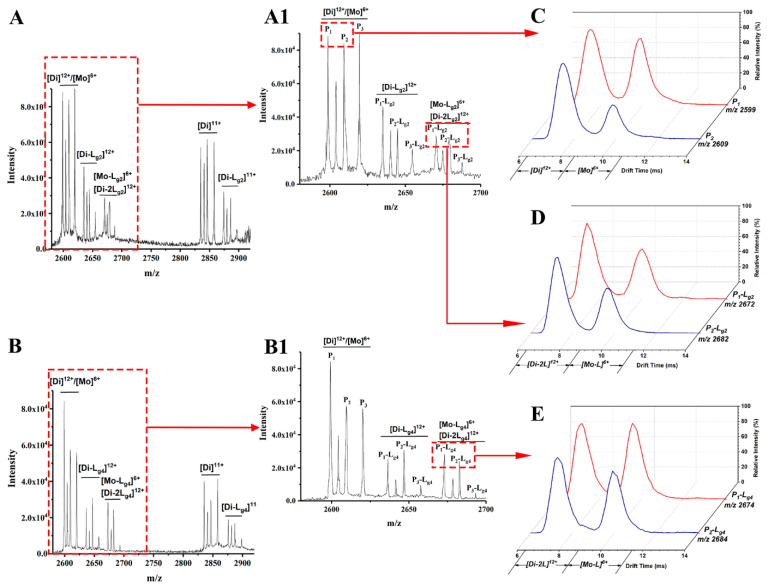
Mass spectra of sophoricoside (L_g2_) with the SOD1 mixture (**A**) and glycitin (L_g4_) with the SOD1 mixture (**B**). An enlarged view between *m/z* 2600 and 2700 is shown in (**A1**,**B1**). The IMS spectra of ApoSOD1 (P_1_) and Cu_2_ SOD1 (P_2_) (**C**), P_1_-L_g2_ and P_2_-L_g2_ complexes (**D**), and P_1_-L_g4_ and P_2_-L_g4_ complexes (**E**) are extracted from the MS peaks of the *m/z* labeled beside the IMS curves.

L_g2_ and L_g4_ are chosen as an example of glycosides interacting with the P_1_–P_3_ mixture. The possible target proteins for these glycosides include P_1_–P_3_ dimers (Di) and the monomers (Mo) dissociated from these dimers. Thus, ion mobility spectrometry (IMS) is used together with ESI-MS to confirm the targets. As shown in MS spectra (Figure 2A,A1), (Figure 2B,B1) of glycosides (L_g2_ and L_g4_) interacted with the P_1_-P_3_ mixture, and complex ions with different charges were found, such as [Di-L]^11+^, [Di-L]^12+^ and [Di-2L]^12+^ or [Mo-L]^6+^, etc. It is found that the intensity of all P_3_-L complex ions is far less than that of those complexes of P_1_-L or P_2_-L. Therefore, P_1_ and P_2_ are regarded as the main binding targets of the glycosides, while P_3_ (Cu_2_Zn_2_SOD1) will no longer be concerned in this study. The 12+ charged dimer ([Di]^12+^) and 6+ charged monomer ([Mo]^6+^) of P_1_ or P_2_ share the same *m*/*z* in ESI-MS spectra (*m*/*z* 2599 for P_1_ and 2609 for P_2_), and can be divided after being extracted into IMS spectra (Figure 2C). According to our previous work, ref. [30] the peak at low drift time corresponds to [Di]^12+^ and the other one corresponds to [Mo]^6+^. Such results also occur for P-L complexes, as shown in Figure 2D,E, which show the extracted IMS spectra from the MS peaks of the complexes including P_1_-L_g2_ at *m*/*z* 2672, P_2_-L_g2_ at m/z 2682, P_1_-L_g4_ at m/z 2674, and P_2_-L_g4_ at m/z 2684. The IMS spectra profile of the complex ions is similar to that of P_1_ and P_2_, which means that their complexes also contain [Di-2L_g_]^12+^and [Mo-L_g_]^6+^ forms. Thus, the two peaks in the IMS spectra of each complex can be classified as [Di-2L]^12+^ and [Mo-L]^6+^. Therefore, both dimers and monomers of P_1_ and P_2_ can be regarded as the main binding targets of ligands. A series of calculations reflecting the complex generation quantity is listed in Table S2. According to the results, the dimer of P_1_ seems to more easily bind with the four ligands. P_2_ complexes also show appreciable percentages. The difference is that the binding rate of the P_2_ monomer seems slightly higher than its dimer. P_3_ shows the lowest binding ability, which is the same as the results of the visual observation of the spectra.

### 2.2. Binding Affinity of ApoSOD1, Cu_2_SOD1 with Glycosides

**Figure 3:**
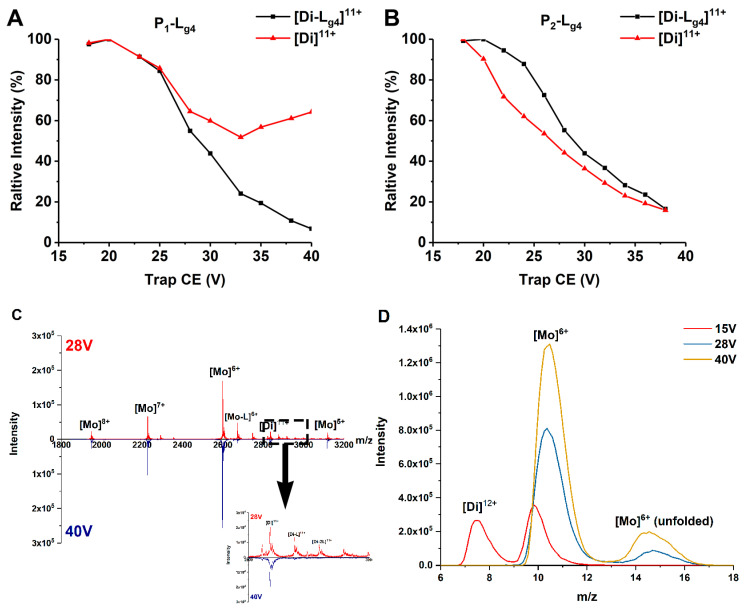
(**A**,**B**) Relative intensity changes of ApoSOD1 and Cu_2_SOD1 (P_1_ and P_2_) and their Di-L_g4_ (protein dimer-glycitin) complexes as trap CE increases. (**C**) The mass spectra of P_1_ at 28 V and 40 V of Trap CE with an enlarged view of the mass range between *m/z* 2800 and 3000. (**D**) The IM spectra of *m/z* 2600 extracted from (**C**) with an additional comparison of the same peak under 15 V of Trap CE.

Exerting collision energy has been found to dissociate SOD1 dimer and SOD1-ligand complexes [13,30]. SOD1-ligand complexes may dissociate by losing a ligand or a subunit in CID. A clear dissociation pathway of SOD1 dimer and SOD1-ligand complexes helps explain and compare the binding affinity of ligands and the gas phase stability of different SOD1s and complexes. Thus, we performed CID experiments on the 11+ charged dimer ions of P_1_, P_2_, and their complexes with L_g1_–L_g4_. The ions are given a collision energy between 15 and 40 V in the trap region before being detected. L_g4_ is chosen as the representative compound to compare the binding affinities with different SOD1s. The dissociation behavior of complexes differs as CE increases. Figure 3 shows the change of relative intensity of the [Di]^11+^ and [Di-L_g4_]^11+^ ions of proteins (P_1_ and P_2_) and their Di-L_g4_ (protein dimer-glycitin) complexes with trap CE. In the P_1_ group (Figure 3A), the relative intensity of the [Di]^11+^ ion of P_1_ dimer is found to decrease as CE grows at a lower CE. This situation is reversed at about 26 V. The relative intensity of [Di]^11+^ ion of the P_1_ dimer comes to a minimum value and then increases. This strongly indicates that the complex (P_1_-L_g4_) dissociates into P_1_ dimer and ligand (L_g4_) at high CE because the P_1_-L_g4_ complex is the only source of the P_1_ dimer that is increasing. If we take the MS spectra of 28 V and 40 V of Trap CE, for example, it can be found that the peaks of the complexes nearly disappear under 40 V but the intensity of [Di]^11+^ is similar as that under 28 V. The IM peaks extracted from the MS peak of the protein (Figure 3D) show that the main products under 28 V or higher CE are mostly at the monomer state. That is to say, the complexes under high Trap CE dissociate into separated proteins and ligands and the protein dimers keep the same charge state as the complexes ([Di]^11+^ for example) or dissociate to monomer ([Di-L]^12+^ to [Mo]^6+^ and L). However, copper recombinant SOD1 provides very different results (Figure 3B). The downtrend of the dimer only becomes slighter at about 30V for P_2_-L_g4_. The convergence of intensity tendency of protein dimer and protein dimer-ligand complexes means that the ligand may interact more strongly with P_2_ than P_1_.

MS/MS experiments of the complexes including P_1_-L_g4_ and P_2_-L_g4_ provide another comparison of these complexes (Figure S2). Given 30 V of Trap CE, the intensity of the P_1_-L_g4_ ion ([Di-L_g4_]^11+^) becomes very weak while the P_1_ dimer ion ([Di]^11+^) maintains the strongest peak. In the P_2_ group, the intensity of the [Di-L_g4_]^11+^ ion stays at a high level. The appearance of the [Di]^11+^ ion of the dimer indicates that losing the ligand from the complex is still the main way of dissociation but at a lower extent than P_1_-L_g4_. This result further indicates that the binding of ligands to P_2_ is stronger than that to P_1_. At the same time, the small intensity of [Mo-L_g4_]^6+^ is also found in the P_2_-L_g4_ group, indicating that a small part of the [Di-L_g4_]^11+^ ion of the P_2_-L_g4_ loses one of the subunits before losing a ligand.

**Figure 4:**
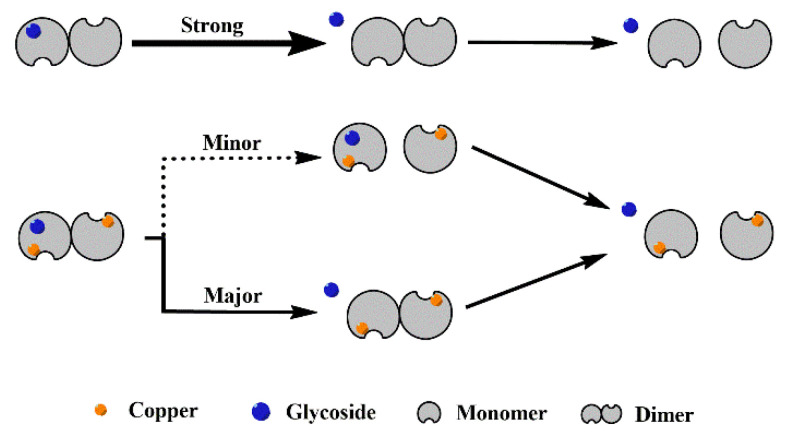
Possible dissociation pathway of the complex ions of different SOD1 (ApoSOD1, Cu_2_SOD1) with glycoside in CID.

Based on the behavior of different SOD1s and their complexes in CID (Figure 3 and Figure S2), it is clear that all the complexes dissociate in the way of losing a ligand, and the recombination of copper considerably enhances the interaction between the four glycosides and SOD1s. With the increasing collision energy, P_1_-L_g_ loses a ligand so easily that the abundance of the free P_1_ dimer can even be increased, as shown in Figure 3A. More affinity of ligand binding taken by copper recombination makes the dissociation slower. As a result, the abundance of the [Di-L_g4_]^11+^ ion of P_2_-L_g4_ can show parallel reduction with the free P_2_ dimer. Figure 4 shows the possible dissociation pathway of the complex ions of different SOD1s (ApoSOD1, Cu_2_SOD1) with glycoside in CID.

### 2.3. Conformation Stability of the Complexes of ApoSOD1 and Cu_2_SOD1 (P_1_ and P_2_) with Glycosides

Collision energy not only dissociates SOD1 dimer or bound ligands but also makes SOD1 unfold [30]. Here we performed a collision-induced unfolding (CIU) experiment by IMS-MS to simulate the unfolding process of abnormal SOD1s and evaluate how the binding of soybean isoflavone glycosides influences the conformation stability of ApoSOD1 and Cu_2_SOD1 (P_1_ and P_2_). As the 6+ charged monomer ion in IMS shows the same drift time as partly unfolded 12+ charged dimer ion, we chose the 11+ charged species as our analysis objects.

**Figure 5:**
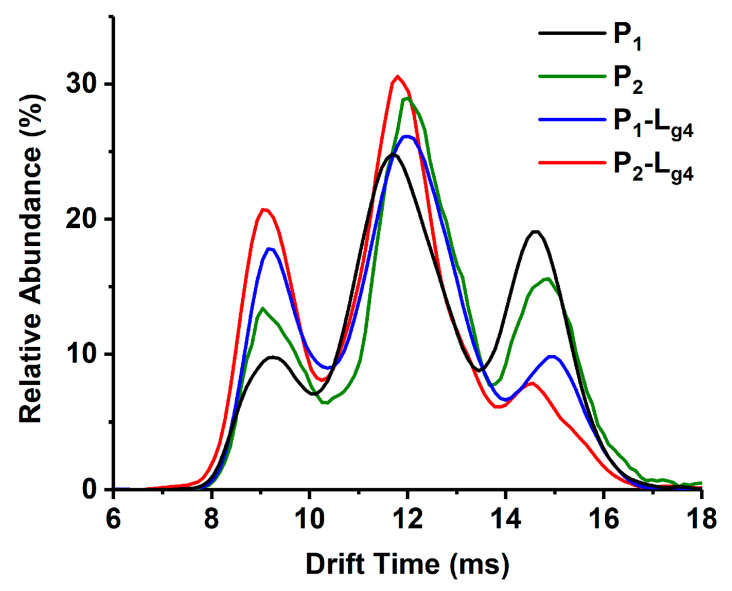
IMS spectra of Apo/Cu_2_SOD1 (P_1_/P_2_) and their complex with glycitin (P_1_/P_2_-L_g4_) at a collision energy of 30 V.

The unfolding of the SOD1 dimer contains three different states: native state (conformation A or Di-A) at low CE, partially unfolded state (conformation B or Di-B), and wholly unfolded state (conformation C or Di-C) [30]. The unfolded state of protein means more risk of aggregation. The unfolded conformation of protein brings a significant growth in collision cross section (CCS). This means higher drift time in IMS if the charge state does not change. Figure 5 shows the relative contents of three conformations of P_1_, P_2_, and P_1_/P_2_-L_g4_ at the CE of 30 V. The binding of the ligand does not bring conformation changes of the three states but rather brings abundance differences. The abundance of compact conformation of the P-L complexes is higher than P and, in turn, P-L complexes unfold less than P. To show the effect on protein conformation from different ligands, the relative abundance of conformation A is read from the IMS spectra and is listed in Table S3. It is found from the P_1_ complexes that L_g4_ as a ligand increases the relative abundance of conformation A by about 5%. L_g2_ and L_g3_ behave less effectively in protecting conformation A. L_g1_ is the least effective in preventing the unfolding of P_1_. For P_2_ complexes, L_g4_ also shows the best in stabilizing the folding structure. L_g1_ still affects little in conformation A.

**Figure 6:**
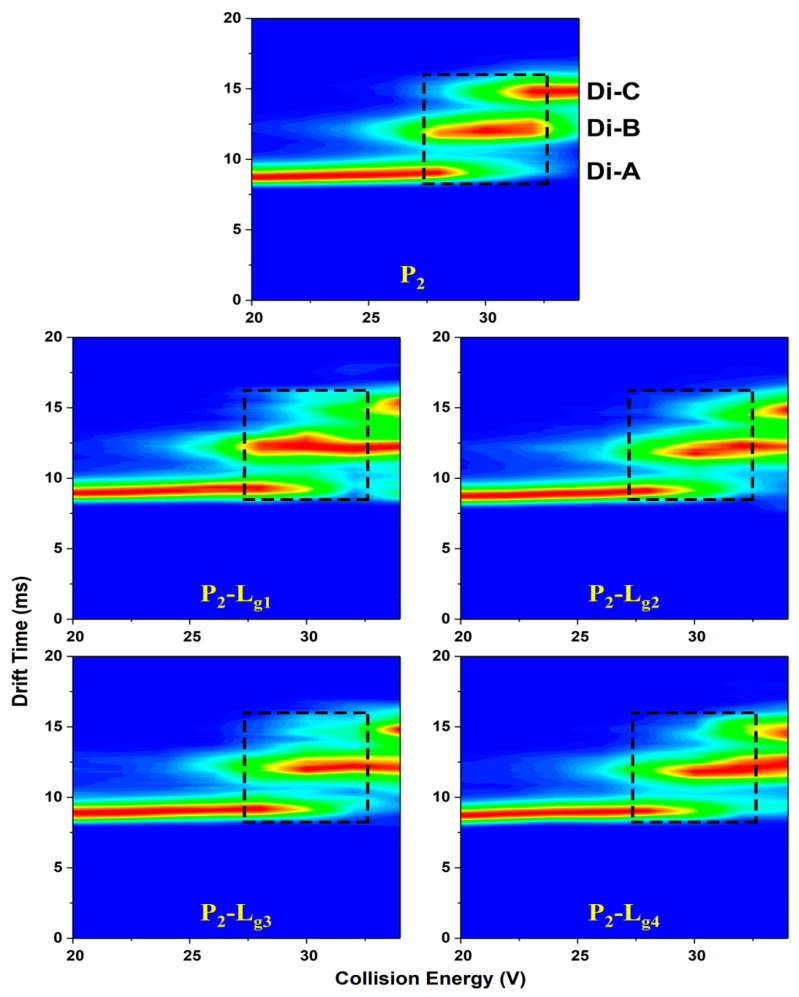
CIU heat maps of Cu_2_SOD1 (P_2_) and its complexes with four glycosides, respectively (P_1_-L_g1_, P_1_-L_g2_, P_1_-L_g3_, and P_1_-L_g4_). The three conformations are marked at the corresponding drift time. The dashed areas of trap collision voltages (27 V to 32 V) show a comparison between protein and complexes on the boundary voltage of conformation conversion.

CIU (collision-induced unfolding) heat maps (Figure 6, Figure S3), matching drift time (DT), collision energy (CE), and relative intensity are plotted to give a comprehensive analysis of the unfolding difference taken by glycosides. The three independent areas represent the DT-CE distribution of the three conformations. The CE region of high intensity (red area) reflects the folding stability from another aspect. It is found that P_2_-L_g_ needs higher CE to convert conformation A into B or B into C (Table S4), which means the glycosides can stabilize the conformation of P_2_ at all unfolding stages. The P_1_-L_g_ only shows a little difference with P_1_.

The results from the heat maps and IMS spectra at the CE of 30 V are unified. It is indicated from both the content of unfolded proteins and conformation conversion CE that the action of L_g2_, L_g3_, and L_g4_ are effective in inhibiting unfolding but different in some aspects. Specifically, the totally unfolded form of the proteins can surely be reduced in varying degrees, decided mainly by the ratio of P-L. L_g4_ shows the strongest ability to keep compact conformation.

### 2.4. The Effects of Soybean Isoflavone Glycosides on the Aggregation of Different SOD1s

**Figure 7:**
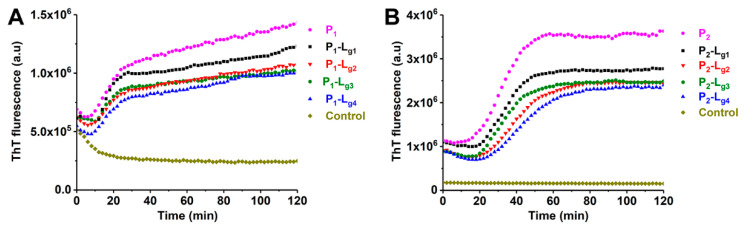
Aggregation kinetics of (**A**) ApoSOD1 (P_1_) and (**B**) Cu_2_SOD1 (P_2_) and their P-L_g_ complexes. The control groups contain all compounds except for proteins.

ApoSOD1 can form toxic amyloid-like aggregates rapidly while being induced by TFE [31,32]. Under the induction of TFE, ApoSOD1 can partly unfold (which occurs at structures such as β-5, β-6) and quickly self-assemble. ThT is a fluorescent indicator which can specifically bind with aggregated β sheets. In this work, the ThT fluorescence intensity of TFE-induced samples was measured continuously to show the kinetics of aggregation. The influence of ligands and copper ions could be evaluated by the change in the aggregation process. The kinetics of P_1_, P_2_, and P_3_ are shown in Figure S4. It can be found that the aggregating speed and extent of P_1_ (ApoSOD1) are much higher than copper recombined SOD1 (Cu_2_SOD1). The results shown in Figure 7 indicate that the four glycosides can inhibit the aggregation of P_1_ and P_2_. Considering the stochastic formation of aggregation [33], L_g2_, L_g3_, and L_g4_ show similar inhibition effects on aggregation and the effects are better than L_g1_. This is reasonable because L_g2_, L_g3_, and L_g4_ were found more effective in stabilizing the folding structure of the target SOD1s than L_g1_ in the CIU results.

### 2.5. Binding Competition of Glycitin and Two Other Small Molecules to Cu_2_SOD1 (P_2_)

**Figure 8:**
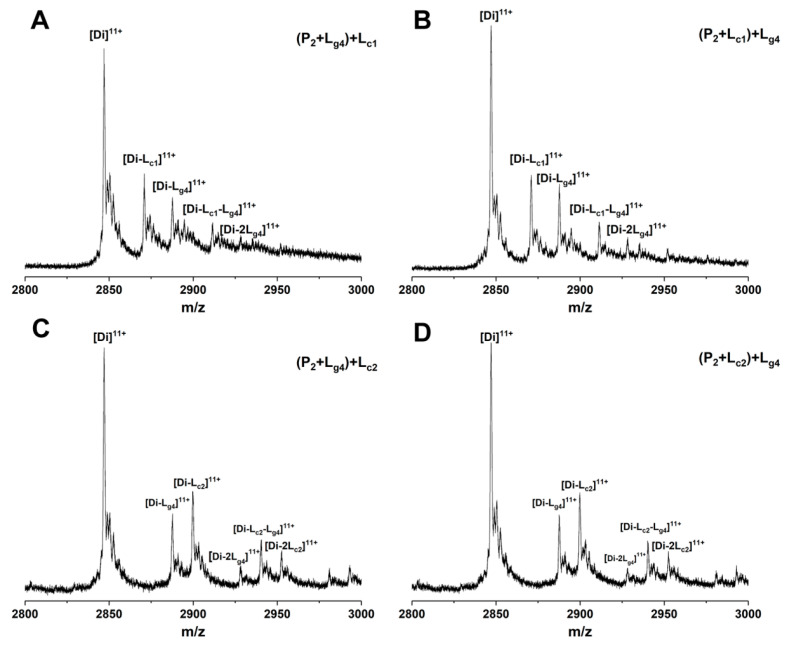
ESI-MS spectra of competition binding of glycitin (L_g4_) with control compound (L_c1_:5-Furd or L_c2_: naringin) to Cu_2_SOD1 (P_2_). The compounds in the bracket are mixed and incubated for 1 h followed by adding the second ligand. Using 5-Furd (L_c1_) as the control compound, the spectra of glycitin (L_g4_) were added, respectively, as the first ligand (**A**) or second ligand (**B**). Using naringin (L_c2_) as the control compound, the spectra of glycitin (L_g4_) were added respectively as the first ligand (**C**) or second ligand (**D**).

The binding sites of some small molecules such as isoproterenol and hesperidin on SOD1 have been predicted experimentally or theoretically [34,35]. Competitive binding between such molecules and other molecules showing binding affinity with unknown sites helps in judging the binding sites [14,36]. 5-Furd (L_c1_) and naringin (L_c2_) were reported as two stabilizers of SOD1 previously [8,13]. L_c1_ was found to bind with SOD1 on the surface of the β-barrel by XRD measurement, which contributes a lot to fibrous aggregation. The results of molecular docking and molecular dynamics simulation in our previous study showed that L_c2_ combines with SOD1 at the dimer interface. In this work, to study whether the soybean isoflavone glycosides would competitively bind with SOD1 against other ligands due to similar binding sites, we used L_c1_ and L_c2_ as control compounds to investigate the competitive binding of glycitin (L_g4_) and control compounds to P_2_. Different ligands were added into samples containing P_2_ in a different order and then analyzed by ESI-MS. Figure 8 shows the ESI-MS spectra of competitive binding of L_g4_ with control compounds (L_c1_ or L_c2_) to P_2_. In the experiments, L_c1_ or L_g4_ was first incubated with P_2_ for 1 h followed by the addition of the other ligand and incubation for another 1 h. Then the samples were subjected to MS measurement. Figure 8A,B shows that the addition of L_g4_ in a different order does not affect the intensity of [Di-L_c1_]^11+^ and [Di-L_g4_]^11+^, while a complex ([Di-L_c1_-L_g4_]^11+^) of two ligands (L_c1_ and L_g4_) bound to P_2_ is found in similar intensity. This result implies that L_g4_ may not bind at the same site as L_c1_. The results of L_c2_ as a control compound are similar to that of L_c1_ above. The spectra of L_g4_ added respectively as the first or second ligand are shown in Figure 8C,D. The intensities of the complexes in Figure 8C,D are similar, including [Di-L_c1_]^11+^, [Di-L_g4_]^11+^, and [Di-L_c1_-L_g4_]^11+^. The result suggests that L_g4_ and L_c2_ may bond at different sites on P_2_. These results indicate that the competition ligands at least do not completely replace the site of the ligand that is added first. Thus, it can be predicted that L_g4_ does not share identical binding sites with either L_c1_ or L_c2_.

### 2.6. Binding Sites of Glycitin Analyzed by Molecular Docking

**Figure 9:**
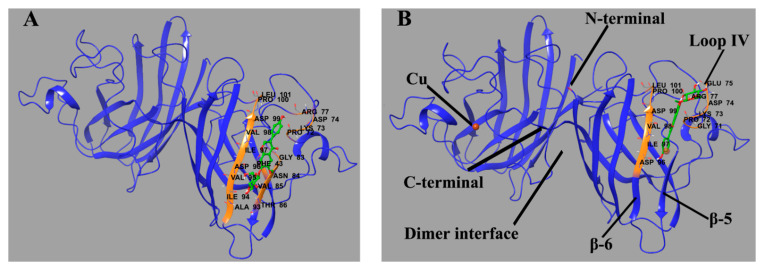
Predicted binding site of glycitin in ApoSOD1 (**A**) (binding energy = −6.33 kcal/mol) and Cu_2_ SOD1 (**B**) (binding energy = −5.41 kcal/mol). The residues that interact with glycitin are labeled and shown as secondary structures (colored) as well as primary structures in the form of sticks. Glycitin is also shown in the form of sticks. The H-bonds between the ligand and residues are shown as green spherical dotted lines.

Molecular docking was performed to predict the binding sites of L_g4_. The sites (Figure 9) in P_1_ and P_2_ with low binding energy are placed at similar residues. Specifically, L_g4_ binds between β-sheet 6 (residue 94–100) and part of loop IV (residue 72–77). The ligand is oriented along the β-sheet and builds a connection between the β-sheet and loop IV through H-bonds. As reported, β-sheet 6 is the core region of aggregation in SOD1 [37,38,39]. Loop IV builds a link between the edge β-sheets and the whole structure and contributes a lot to dimerization and the enzymatic activity of SOD1 [40]. It is quite rigid in Cu_2_Zn_2_SOD1 but highly flexible in some SOD1 mutants. These fragments in some SOD1 mutants can be more unstable and easily unfold while being induced. Therefore, the binding of glycitin provides additional intermolecular interaction to stabilize the conformation of SOD1 as well as prevent aggregation.

### 2.7. Calculation and Comparison of Collision Cross Section

Collision cross section (CCS) is usually used to evaluate the stretch degree of proteins. It can be measured by IMS and calculated through a theoretical model. In this study, the CCS of P_1_ and P_2_ and their L_g4_-bound complexes are given by two methods: measured CCS from standard lines (Figure S5) and drift times of target components and theoretical CCS calculated through the computational method [41]. All results are listed in Table 1 and Table 2. As the CCS of all species has little difference, the recombination of metal ions and glycitin makes little effect on the size of the overall structure of SOD1. It can be inferred from these results that losing metal ions or other stabilizers may not make SOD1 unfold immediately. However, SOD1 without metal ions or stabilizers can hardly stay stable in dynamic processes or external induction such as the collision energy in MS or TFE-induced aggregation.

## 3. Materials and Methods

### 3.1. Materials

Cu_2_Zn_2_SOD1 from bovine erythrocytes was purchased from Beyotime Biotechnology (Shanghai, China). Daidzein, genistein, sophricoside, genistin, and (purity ≥98% for all) were provided from Nature Institutes for Food and Drug Control(Beijing, China). Glycitein, daidzin, and flycitin were purchased from Chengdu Herbpurify Co., LTD (Chengdu, China). Horse cytochrome c, horse myoglobin, naringin, Thioflavin-T (ThT), ammonium acetate, and 5-furd were obtained from Sigma-Aldrich (St. Louis, MO, USA). Trifluoroethanol (TFE) was purchased from J&K Scientific Ltd. (Beijing, China). Ethylenediaminetetraacetic acid (EDTA) and copric chloride dihydrate (CuCl_2_.2H_2_O) were obtained from Beijing Chemical Works (Beijing, China). Methanol and formic acid were supplied by TEDIA Company (Fairfield, OH, USA). The ultrapure water used in all experiments was prepared by a Milli-Q water purification system (Milford, MA, USA). The dialysis devices (Micro Float-A-Lyzer, MW-cut: 10 kDa) were bought from Spectrum Laboratories (Rancho Dominguez, CA, USA). The isoflavones, 5-furd, and naringin were dissolved at 1 mM in methanol as stock solutions and stored at 4 °C before experiments.

### 3.2. Preparation of ApoSOD1 and Cu Recombined SOD1

Step 1: Cu_2_Zn_2_SOD1 was first dialyzed by Float-A-Lyzer G2 (10-kDa MW-cut) in a 20 mM ammonium acetate buffer (pH = 3.2) containing 5 mM EDTA for 24 h.

Step 2: The buffer was changed into 20 mM ammonium acetate (pH = 3.2) to remove EDTA.

Step 3: The buffer differs for ApoSOD1 and Cu_2_SOD1. A 20 mM ammonium acetate buffer (pH = 6.8) was used to refold ApoSOD1 while a 20 mM ammonium acetate buffer (pH = 3.2) containing 5 mM CuCl_2_ for Cu_2_SOD1. ApoSOD1 was prepared and collected after 24 h of dialysis followed by ultrafiltration. For Cu_2_SOD1, the time of dialysis was usually about 4–6 h.

Steps 4 and 5 are only for Cu_2_SOD1.

Step 4: The buffer was changed into 20 mM ammonium acetate (pH = 3.2). The dialysis lasted for 6 h.

Step 5: The buffer was changed into 20 mM ammonium acetate (pH = 6.8). The dialysis lasted for more than 12 h followed by ultrafiltration and product collection.

Step 6: For both products, 10 μL of the product was diluted at 10 mM ammonium acetate (pH = 6.8) to 200 μL. Then the samples were analyzed by ESI-MS after ultrafiltration to confirm the metal content. The products with unexpected metal content would be re-dialyzed from the first step.

The mass spectra of products suitable for other experiments are shown in Figure S6.

### 3.3. Mass Spectrometry Experiments

For all MS samples, the proteins and ligands were mixed and diluted into 5 μM and 30 μM by 10 mM ammonium acetate (pH = 6.8). The samples were incubated at 37 °C for 1 h before analysis.

The MS experiments were all performed on a quadrupole ion-mobility time-of-flight (Q-IM-TOF) mass spectrometer (Synapt G2-S, Waters Corp., Manchester, UK). Samples were directly infused at a flow rate of 10 μL/min and detected under positive-ion mode. If not mentioned specifically, all the conditions were set at optimized values. The capillary voltage was set at 2.40 kV and the cone voltage at 40 V. The source temperature was kept at 80 °C. As the desolvation gas, we used nitrogen at a flow rate of 400 L/h under 150 °C. Nitrogen was also the IMS gas in the IMS experiments and its flow rate was 90.00 mL/min. At the same time, manual control of IMS parameters was switched on for optimization. As a result, IMS wave velocity was set to 600 m/s and IMS wave height to 33.0 V. All data were acquired and analyzed by Masslynx 4.1 software (Waters Corp., Manchester, UK).

### 3.4. Analysis of Aggregation Kinetics

To prepare the samples of ThT fluorescence assays, the protein with or without a ligand was diluted with an ammonium acetate buffer (pH = 6.8) at 50 mM. The concentration of protein and ligand was 15 μM and 150 μM. ThT was added to be 25 μM at the same time. After incubating the samples at 37 °C for 1 h, TFE was added to induce the aggregation at the final concentration of 12%. The samples were quickly mixed well by vortex and transferred into a black 96-well plate. The fluorescence was measured every 2 min by a Molecular Devices Spectra Max i3x instrument. The excitation and emission wavelengths were set at 440 and 485 nm.

### 3.5. Competition Binding Experiments of Glycitin with Control Compounds to Cu_2_SOD1

The protein and the first ligand were mixed and diluted into 5 μM and 30 μM by 10 mM ammonium acetate (pH = 6.8) and incubated at 37 °C for 1 h. Then the second ligand was added, and the concentration was also 30 μM. After incubation for another 1 h, the samples were analyzed by ESI-MS. The method and instrument parameters are the same as in other ESI-MS experiments.

### 3.6. Molecular Docking

The structure of bovine SOD1 (PDB code 1SXA [42,43]) was prepared using Autodock 4.2.6 program [44]. Water and metal ions (all ions for ApoSOD1 and zinc ions for Cu_2_ SOD1) were removed from the models and saved as rigid macromolecules for docking. The force field parameters of copper ions were manually into the configuration files of the program for distinguishing the copper ions in Cu_2_SOD1. The 3D structure of glycitin was downloaded from the PubChem website and optimized in the Autodock program. For docking parameters, glycitin was set as a flexible ligand for semiflexible docking. The grid box was set as 80 × 108 × 70 and was centered in one of the subunits of SOD1 to include the whole subunit and dimer interface. The binding models were output using the Lamarckian Genetic Algorithm and sorted by binding energy. Reasonable docked structures with the lowest binding energy were chosen as the final results.

### 3.7. Measurements and Calculation of Collision Cross Section

Theoretically, the calibration drift time (t^’^_d_) and CCS (Ω_c_) have a relationship, as Formula (1) shows. The calibration drift time and CCS can be calculated by Formulas (2) and (3) [45,46,47]. Here, t_d_ represents IMS measured drift time and Ω means the actual CCS. M_G_ means the relative molecular mass of IMS gas. The drift time (t_d_) of cytochrome c and myoglobin under six different IMS parameters was measured first. The CCS (Ω) of these two standard proteins was provided by the CCS database. Then, linear fitted curves were plotted according to Formula (1). The drift time of SOD1-involved samples was measured under the same IMS parameters. The results were substituted into the formulas to get the measured CCS.

ln(Ω_c_) = Xln(t^’^_d_) + A (1)

t’_d_ = t_d_ − 1.57 × 10^−3^ × (*m*/*z*)^0.5^ (2)

Ω_c_ = Ω/z[(m + M_G_)/mM_G_]^0.5^ (3)

For the calculation of theoretical CCS, Collidoscope [41], an open source program, was employed to calculate the CCS of SOD1 or ligand-bound SOD1. Necessary modification for molecule models was performed before calculation. The calculation was processed using a charge placement algorithm. The net charge was set to be 11 and the collision gas was set as spherical N_2_. As there is a lack of Lennard-Jones parameters of Cu in the default files, all Cu atoms in Cu_2_SOD1-involved molecules were deleted as the last modification step for the models.

## 4. Conclusions

In summary, we screened four glycosides from seven soybean isoflavones showing stable non-covalent binding affinity with SOD1 at three different metallization states. The results of CID-MS and MS/MS indicated that the recombination of copper played an important role in increasing the binding affinity of the four glycosides. This may be the result from the stabilization effect of copper according to other experiments and calculations. CIU-IMS-MS showed that ApoSOD1 and Cu_2_SOD1 need higher collision energy to unfold after binding with the glycosides. TFE-induced aggregation kinetics experiments showed that both Cu^2+^ and the four glycosides had significant effects on the formation of aggregates. As seen from the results of MS and aggregation kinetics, glycitin showed the best effect in stabilizing the SOD1 structure and inhibiting its aggregation, followed by sophoricoside and genistin. Competitive interaction experiments between two small molecules whose binding sites in SOD1 were known and glycitin were performed, and we made sure that these ligands shared different binding sites. Molecular docking also supported this result and showed that glycitin interacts with SOD1 at β-sheet 6 and loop IV. In conclusion, soybean isoflavone glycosides were hopeful small molecules in inhibiting the aggregation of abnormal metalized SOD1.

## Figures and Tables

**Table 1:** IMS measured CCS (nm^2^) of ApoSOD1 and Cu_2_ SOD1 (P_1_, P_2_) and complexes with glycitin (P_1_-L_g4_, P_2_-L_g4_) under six IM wave velocities (WV) and wave heights (WH).

WV(m/s)/WH(V)	430/40	350/40	400/40	430/35	400/37	360/37
P_1_	23.82	24.01	23.22	24.62	24.16	23.77
P_1_-L_g4_	24.02	24.23	23.55	25.89	24.33	23.95
P_2_	23.50	25.19	23.64	22.97	24.43	23.11
P_2_-L_g4_	23.68	25.35	23.81	23.04	24.57	23.28

**Table 2:** Average measured CCS (nm^2^) and theoretically calculated CCS (nm^2^) of ApoSOD1(P_1_) and Cu_2_SOD1(P_2_) and their complexes (P_1_-L_g4_, P_2_-L_g4_). The error of measured CCS is the standard deviation of the CCS measured under six parameters (see Table 1). The error of calculated CCS is the standard deviation of the CCS calculated under different energy states (*n* = 4).

	IMS Measured	Theoretically Calculated
P_1_	23.93 ± 0.46	26.48 ± 0.11
P_1_-L_g4_	24.33 ± 0.81	26.61 ± 0.10
P_2_	23.81 ± 0.85	26.84 ± 0.16
P_2_-L_g4_	23.96 ± 0.86	26.86 ± 0.18

## Data Availability

The data presented in this study are available in this article and Supplementary Materials.

## Supplementary Materials

The following supporting information is available online on the following link https://www.mdpi.com/article/10.3390/molecules27217303/s1, Table S1: The intensity and fading rate of MS peaks of isoflavone aglycones (La1–La3) and glycosides (Lg1–Lg5) before (I) and after (I’) adding SOD1 mixture. All compounds were analyzed under ESI negative mode. Figure S1: Intensity fading mass spectra of seven soybean isoflavones before (A) and after (B) adding P1, P2, and P3 mixture. All peaks represent [M-H]- ions of corresponded compounds. Table S2: The relative abundance of complexes compared with the protein species at the same charge state. The percentages (P) of dimer (Di)-ligand complexes were calculated directly through MS intensity (I) of +11 charged complexes and proteins. The formula is P = I (complex)/I(protein). The percentages (P) of monomer (Mo)-ligand complexes were calculated by the integrated IM peak area (A) of +6 charged monomers. The formula is P = A (complex)/A(protein). Figure S2: MS/MS spectra of Apo/ Cu2 SOD1-glycitin (P1-Lg4 and P2-Lg4) complexes. The Trap collision energy is set as 30 V. The precursor ions are the 11+ charged ions of dimer of the complexes ([Di-Lg4]^11+^). Table S3. The relative abundance (%) of conformation A of different SOD1s and complexes. Figure S3: CIU heat maps of ApoSOD1 (P1) and complexes with four glycosides (Lg1-Lg 4). The three conformations are marked at corresponding drift time. Dashed areas of trap collision voltages (26 V to 32 V) show comparison between protein and complexes on the boundary voltage of conformation conversion. Table S4. Conversion CE (V) of Apo and Cu2SOD1(P1, P2) and their complexes with four glycosides (Lg1–Lg4). Figure S4. ThT fluorescence of ApoSOD1 (P1, red), Cu2SOD1 (P2, black), and Cu2Zn2SOD1 (P3, blue) induced by TFE. Figure S5: The linear fitted curves under six IM parameters (wave velocity and wave height) according to Formulas (1), standard CCS and measured drift time of cytochrome c and myoglobin. Figure S6: The MS spectra of ApoSOD1 (left) and Cu_2_SOD1 (right) products after dialysis.

## Sample Availability

Not available.
