# Supplementary material for: Native Mass Spectrometry Coupled to Spectroscopic Methods to Investigate the Effect of Soybean Isoflavones on Structural Stability and Aggregation of Zinc Deficient and Metal-Free Superoxide Dismutase
Source: Molecules. 2022 Oct 27;27(21):7303. doi: 10.3390/molecules27217303 (PMC9654870; doi:10.3390/molecules27217303)
Supplement: Supplementary file 1 [file molecules-27-07303-s001.zip › molecules-1959333-supplementary.pdf]

Native mass spectrometry coupled to spectroscopic methods to investigate the effect of soybean isoflavones on structural stability and aggregation of zinc deficient and metal-free superoxide dismutase

Xinyu Bian[1, 2], Xiaoyu Zhuang[3], Junpeng Xing[1], Shu Liu[1, *], Zhiqiang Liu[1], Fengrui Song[1, 2, *]

[1] State Key Laboratory of Electroanalytical Chemistry, Jilin Province Key Laboratory of Chinese Medicine Chemistry and Mass Spectrometry, Changchun Institute of Applied Chemistry, Chinese Academy of Sciences, Changchun, 130021, China. mslab9@ciac.ac.cn (X.B.); xyzhuang59@foxmail.com (X.Z.) xjp@ciac.ac.cn (J.X.); liuzq@ciac.ac.cn (Z.L.)

[2] School of Applied Chemistry and Engineering, University of Science and Technology of China, Hefei, 230029, China

[3] Experiment Center for Science and Technology, Shanghai University of Traditional Chinese Medicine, Shanghai, 201203, China

* Correspondence: mslab20@ciac.ac.cn (S.L.) and songfr@ciac.ac.cn (F.S.); Tel.: +86-431-85262044

Table S1. The intensity and fading rate of MS peaks of isoflavone aglycones (L_{a1}- L_{a3}) and glycosides (L_{g1}-L_{g5}) before (I) and after (I') adding SOD1 mixture. All compounds were analyzed under ESI negative mode.

Compounds (m/z)	I	I'	(I-I')/I×100%
L_{a1} (253.03)	8.60×10^6	8.51×10^6	1.05%
L_{a2} (269.02)	6.40×10^6	6.35×10^6	0.78%
L_{a3} (283.03)	5.32×10^6	5.26×10^6	1.13%
L_{g1} (415.08)	4.19×10^6	3.37×10^6	19.57%
L_{g2}+L_{g3} (431.07)	1.12×10^7	9.48×10^6	15.36%
L_{g4} (445.08)	2.47×10^6	1.81×10^6	26.72%

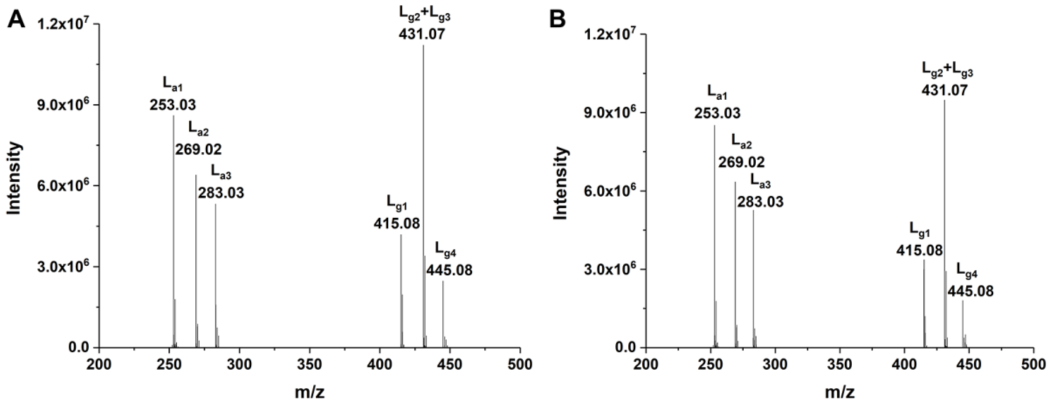

Figure S1. Intensity fading mass spectra of seven soybean isoflavones before (A) and after (B) adding P_1, P_2, and P_3 mixture. All peaks represent $[M-H]^-$ ions of corresponded compounds.

Table S2. The relative abundance of complexes compared with the protein species at the same charge state. The percentages (P) of dimer (Di)-ligand complexes were calculated directly through MS intensity (I) of +11 charged complexes and proteins. The formula is P=I (complex)/I(protein). The percentages (P) of monomer (Mo)-ligand complexes were calculated by the integrated IM peak area (A) of +6 charged monomers. The formula is P=A (complex)/A(protein).

**Compounds**	**Apo**		**Cu_2**		**WT**	
	**Mo**	**Di**	**Mo**	**Di**	**Mo**	**Di**
**Daidzin**	56.13%	87.22%	39.60%	38.01%	24.84%	24.13%
**Sophoricoside**	25.18%	56.85%	27.93%	22.16%	13.90%	19.29%
**Genistin**	42.35%	58.62%	49.73%	32.68%	21.48%	24.54%
**Glycitin**	48.96%	50.17%	23.47%	22.26%	16.41%	17.06%

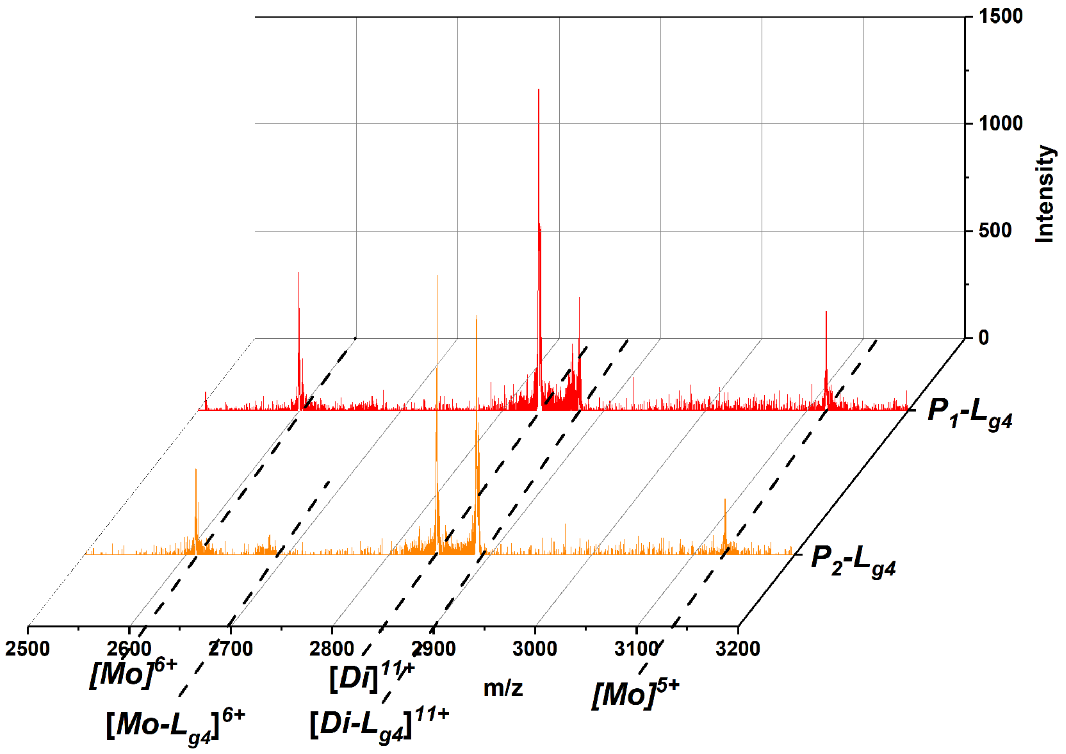

Figure S2. MS/MS spectra of Apo/ Cu_2 SOD1-glycitin (P_1 -L_{g4} and P_2-L_{g4}) complexes. The Trap collision energy is set as 30V. The precursor ions are the 11+ charged ions of dimer of the complexes ($[Di\text{-}L_{g4}]^{11+}$).

Table S3. The relative abundance (%) of conformation A of different SOD1s and complexes.

	L_{g1}	L_{g2}	L_{g3}	L_{g4}	No ligand
P_1	8.25	11.58	11.22	14.63	9.77
P_2	14.31	18.01	16.33	20.70	13.41

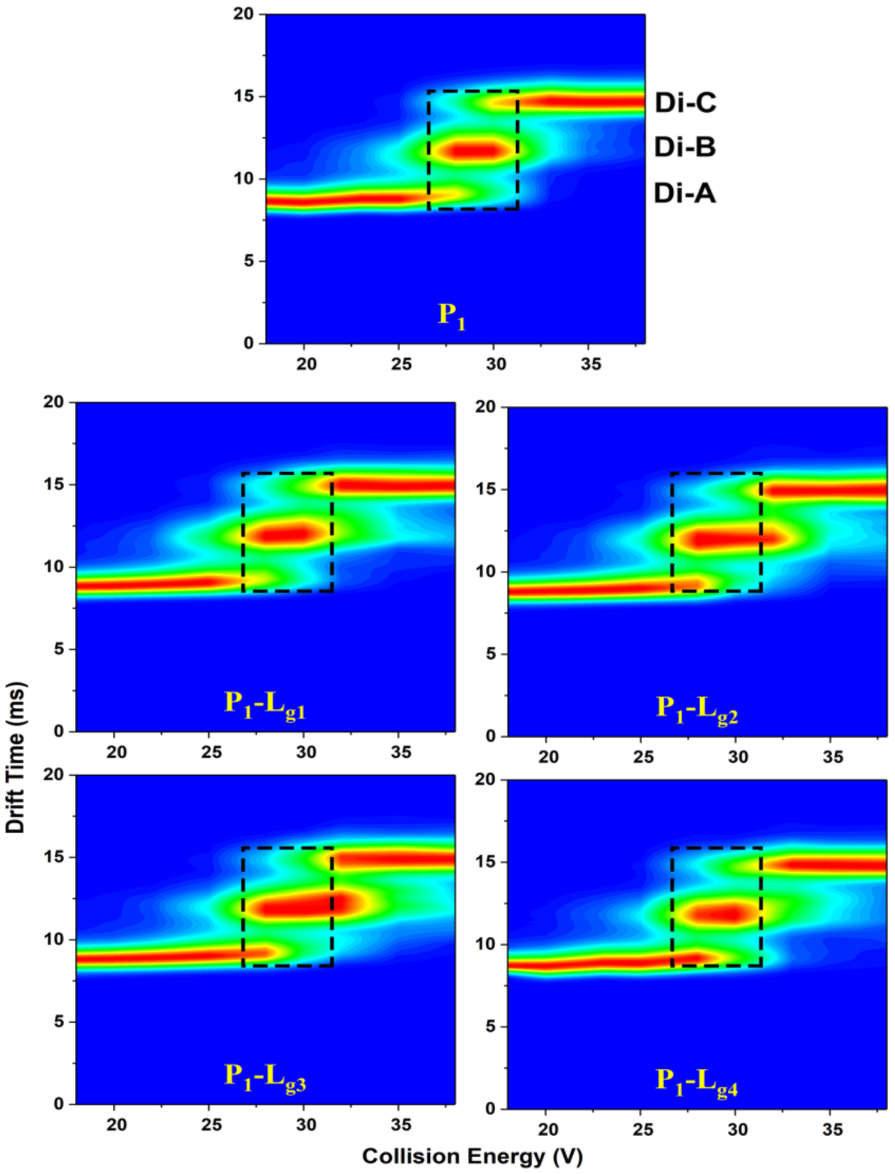

Figure S3. CIU heat maps of ApoSOD1 (P_1) and complexes with four glycosides (L_{g1}-L_{g4}). The three conformations are marked at corresponding drift time. Dashed areas of trap collision voltages (26 V to 32 V) show comparison between protein and complexes on the boundary voltage of conformation conversion.

Table S4. Conversion CE (V) of Apo and Cu_2SOD1(P_1, P_2) and their complexes with four glycosides (L_{g1}-L_{g4}).

Conversion CE (V) of conformation A to B					
	L_{g1}	L_{g2}	L_{g3}	L_{g4}	No ligand
P_1	27	27	27	27	27
P_2	28	29	29	29	30
**Conversion CE (V) of conformation B to C**					
	L_{g1}	L_{g2}	L_{g3}	L_{g4}	No ligand
P_1	30	31	31	31	30
P_2	34	34	34	35	31

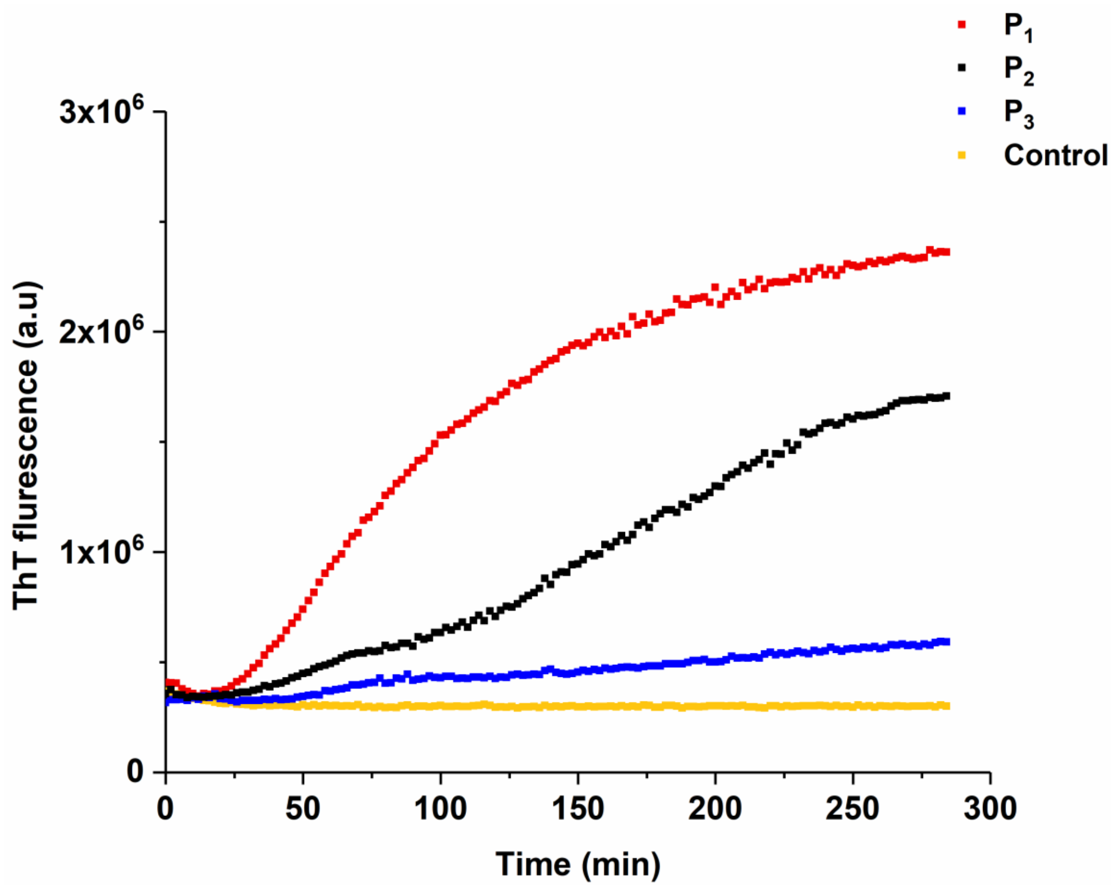

Figure S4. ThT flurescence of ApoSOD1 (P_1, red), Cu_2SOD1 (P_2, black) and Cu_2Zn_2SOD1 (P_3, blue) induced by TFE.

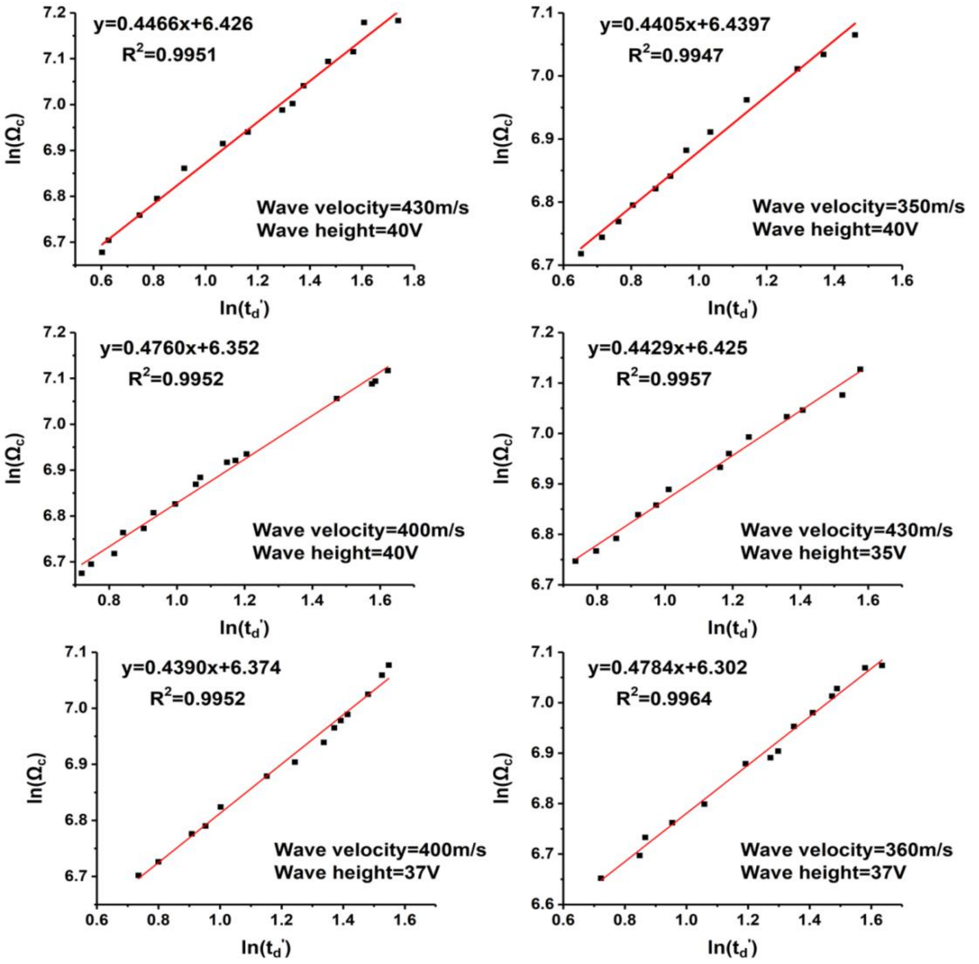

Figure S5. The linear fitted curves under six IM parameters (Wave velocity and Wave height) according to fomula (1) (2), standard CCS and measured drift time of cytochrome c and myoglobin.

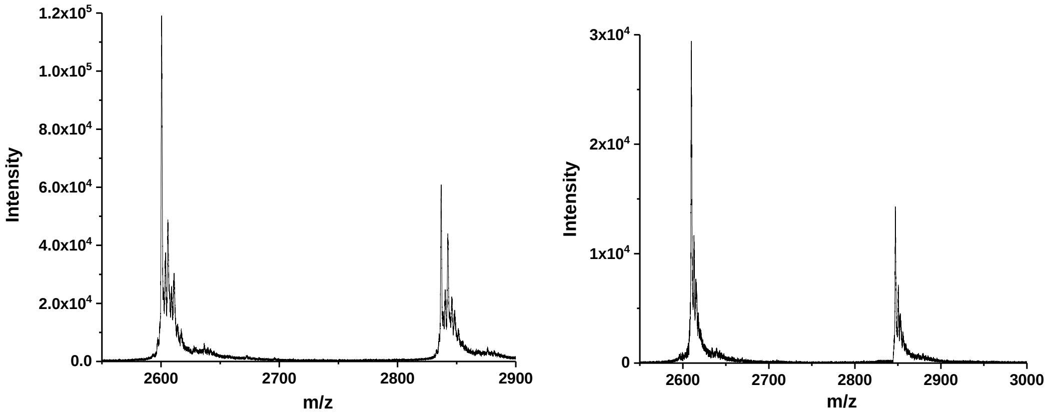

Figure S6. The MS spectra of ApoSOD1 (left) and Cu_2SOD1 (right) products after dialysis.
